# A comparison of maxillofacial growth in Chinese children with isolated cleft palate treated with two different palatoplasty techniques without relaxing incisions: a preliminary study

**DOI:** 10.1186/s12903-023-03588-6

**Published:** 2023-11-23

**Authors:** Sadam Ahmed Elayah, Jiayi Yin, Waseem Saleh Al-Gumaei, Hamza Younis, Karim Ahmed Sakran, Ziwei Tang, Mubarak Ahmed Mashrah, Grace Paka Lubamba, Min Wu, Yang Li, Bing Shi

**Affiliations:** 1 State Key Laboratory of Oral Diseases & National Center for Stomatology & National Clinical Research Center for Oral Diseases and Department of Oral and Maxillofacial Surgery, West China Hospital of Stomatology, Sichuan University, ChengduSichuan, 610041 China; 2 Department of Oral and Maxillofacial Surgery, College of Dentistry, Ibb University, Ibb, Yemen; 3 State Key Laboratory of Oral Diseases & National Center for Stomatology & National Clinical Research Center for Oral Diseases and Department of Orthodontics, West China Hospital of Stomatology, Sichuan University, Sichuan, 610041 China

**Keywords:** Cleft Palate, Sommerlad palatoplasty, Relaxing incisions, Maxillofacial growth

## Abstract

**Objective:**

To assess the maxillofacial growth of patients with isolated cleft palate following the Sommerlad-Furlow modified technique and compare it with the effect of the Sommerlad technique.

**Study design:**

A Retrospective Cohort Study.

**Methods:**

A total of 90 participants, 60 patients with non-syndromic isolated soft and hard cleft palate (ISHCP) underwent primary palatoplasty without relaxing incision (30 patients received the Sommerlad-Furlow modified (S-F) technique and 30 received Sommerlad (S) technique). While the other 30 were healthy noncleft participants with skeletal class I pattern (C group). All participants had lateral cephalometric radiographs at least 5 years old age. All the study variables were measured by using stable landmarks, including 11 linear and 9 angular variants.

**Results:**

The means age at collection of cephalograms were 6.03 ± 0.80 (5–7 yrs) in the S group, 5.96 ± 0.76 (5–7 yrs) in the S-F group, and 5.91 ± 0.87 (5–7 yrs) in the C group. Regarding cranial base, the results showed that there were no statistically significant differences between the three groups in S–N and S–N-Ba. The S group had a significantly shortest S-Ba than the S-F & C groups (*P* = 0.01), but there was no statistically significant difference between S-F and C groups (*P* = 0.80). Regarding skeletal maxillary growth, the S group had significantly shorter Co-A, S- PM and significantly less SNA angle than the C group (*P* =  < 0.01). While there was no significant difference between S-F & C groups (*P* = 0.42). The S group had significantly more MP-SN inclination than the C group (*P* =  < 0.01). Regarding skeletal mandibular growth, there were no statistically significant differences in all linear and angular mandibular measurements between the three groups, except Co-Gn of the S group had a significantly shorter length than the C group (*P* = 0.05). Regarding intermaxillary relation, the S-F group had no significant differences in Co-Gn—Co-A and ANB as compared with the C group. The S group had significantly less ANB angle than S-F & C groups (*P* = 0.01 & *P* =  < 0.01). In addition, there were no significant differences in all angular occlusal measurements between the three groups.

**Conclusion:**

As a preliminary report, Sommerlad-Furlow modified technique showed that maxillary positioning in the face tended to be better, and the intermaxillary relationship was more satisfactory than that in Sommerlad technique when compared them in healthy noncleft participants.

**Supplementary Information:**

The online version contains supplementary material available at 10.1186/s12903-023-03588-6.

## Introduction

The cleft palate is the most frequent birth abnormality overall and the most common developmental deformity in the craniofacial region [1–3]. It has a significant socioeconomic and psychological impact on patients and their families [4, 5]. Palatoplasty has advanced further than just closing the gap to properly functioning reconstruction of the palate with minimal influence on maxillofacial growth in recent years [6]. Although numerous techniques for cleft palate repair have been established, there is no agreement on the ideal palatoplasty technique for all cleft palate types [7–9]. The ideal surgical outcomes of a palate repair should include disconnection of the oral and nasal cavities and competent velopharyngeal closing for speech recovery while maintaining the normal potential growth in the relevant region [10]. No general agreement exists on what causes maxillary growth restrictions in cleft palate patients following primary palatoplasty. No scientific evidence correlates growth restriction with any of its putative factors [11–13]. Maxillofacial growth was reported to be inhibited following V–Y pushback and von Langenbeck approaches [14, 15], and the disruption of potential growth is mostly attributed to the denuded bone as resulting of relaxing incisions left for secondary intent healing [7, 16–19] Numerous animal studies have shown that denudating the palatal bone by the relaxing incision impairs maxillary growth. Techniques without relaxing incisions have less potential to affect maxillary growth adversely when compared with other techniques with relaxing incisions [20–23]. Maxillary dysgenesis is thought to be influenced by scar tissue that forms in the denuded bone region following palate formation. Recent palatoplasty techniques have been developed to limit the impact of this scarring by minimizing the denuded bone [24]. Therefore, there has been a tendency toward emphasizing palatoplasty techniques that avoid relaxing incisions on the hard palate in a functional cleft palate repair [25, 26]. But, considering that there is no relaxing incision, the number of approaches for wide cleft repair will probably be limited. While Sommerlad palatoplasty can improve the function of the palate, there is some debate over its effect on maxillofacial growth.

To expand the surgical indication for palatoplasty without relaxing incision to include wider clefts, we at West China Hospital of Stomatology developed a novel palatoplasty technique called the Sommerlad-Furlow modified palatoplasty (S-F) technique, which involves the most advantageous features of the Sommerlad technique (radicle muscular dissection) and the Furlow technique (Z- plasty). Recently, we explored the incidence of postoperative complications following the S.F technique, including oronasal fistula, velopharyngeal insufficiency, and inadequate quality of life [6, 8, 9, 27]. Meanwhile, the influence of the S-F technique on maxillofacial growth remains unknown [6, 8, 27]. Thus, the current study is the first long-term study that aimed to assess the maxillofacial growth of patient with isolated cleft palate following the S-F technique and compare it with the effect of Sommerlad technique.

## Materials and methods

### Subjects

A retrospective study was conducted on 90 participants, 60 patients with non-syndromic isolated soft and hard cleft palate (ISHCP) who underwent primary palatoplasty without relaxing incision (30 patients received S-F technique and 30 received S technique). While the other 30 were normal participants with skeletal class I pattern.

Two highly experienced cleft surgeons trained by the same surgeon (Shi Bing) performed all cleft palate repairs. They all worked as a team at the same centre, the West China Stomatology Hospital Sichuan University, from 2011 to 2021. Patients were selected based on the subsequent inclusion criteria; Han Chinese patients with nonsyndromic ISHCP who underwent primary palatoplasty by either S-F technique or S technique without relaxing incision within 1–1.6 years old, patients who had lateral cephalometric imaging at least five years following a palatoplasty [5, 28, 29], patients who had not undergone any other surgery besides palatoplasty as Cheiloplasty, Rhinoplasty or, preoperative or postoperative orthodontic treatment, no history of other types of congenital malformation. However, patients with relaxing incisions on the hard palate and secondary palate repair were excluded. The study protocol was reviewed and approved by the Research Subject Review Board and Ethical Scientific Board of Sichuan University (No. WCHS-CRSE-2023–113-R2-P). As well as, it has been conducted by the guidelines of the Declaration of Helsinki. Each of their parents had given informed consent. The control (C) group was matched with both study (S-F & S) groups in number, age, and gender (Table 1).

**Table 1:** Demographic features of participants of groups

Variables	Sommerlad group	S.F group	Control group	*P-value*
**Gender**, #30				
Male	14	15	16	0.76
Female	16	15	14	
**Age at cephalograms collection**, year				
Mean ± SD	6.03 ± 0.80	5.96 ± 0.76	5.91 ± 0.87	0.83
(Min–Max)	(5–7)	(5–7)	(5–7)	

*S-F* Sommerlad-Furlow modified technique, *SD* Standard deviation

### Sample size calculation

The G*power 3.0.10 software was used to calculate the sample size. An effect size of 0.39 was obtained from a previous study [28] for the outcome of S–N between three groups after palatoplasty. The power of the study was set at 0.85, and the alpha error (p-value) was set at 0.05. In addition, it was conducted based on previous comparable studies [5, 30, 31].

### Surgical technique

**Fig. 1:**
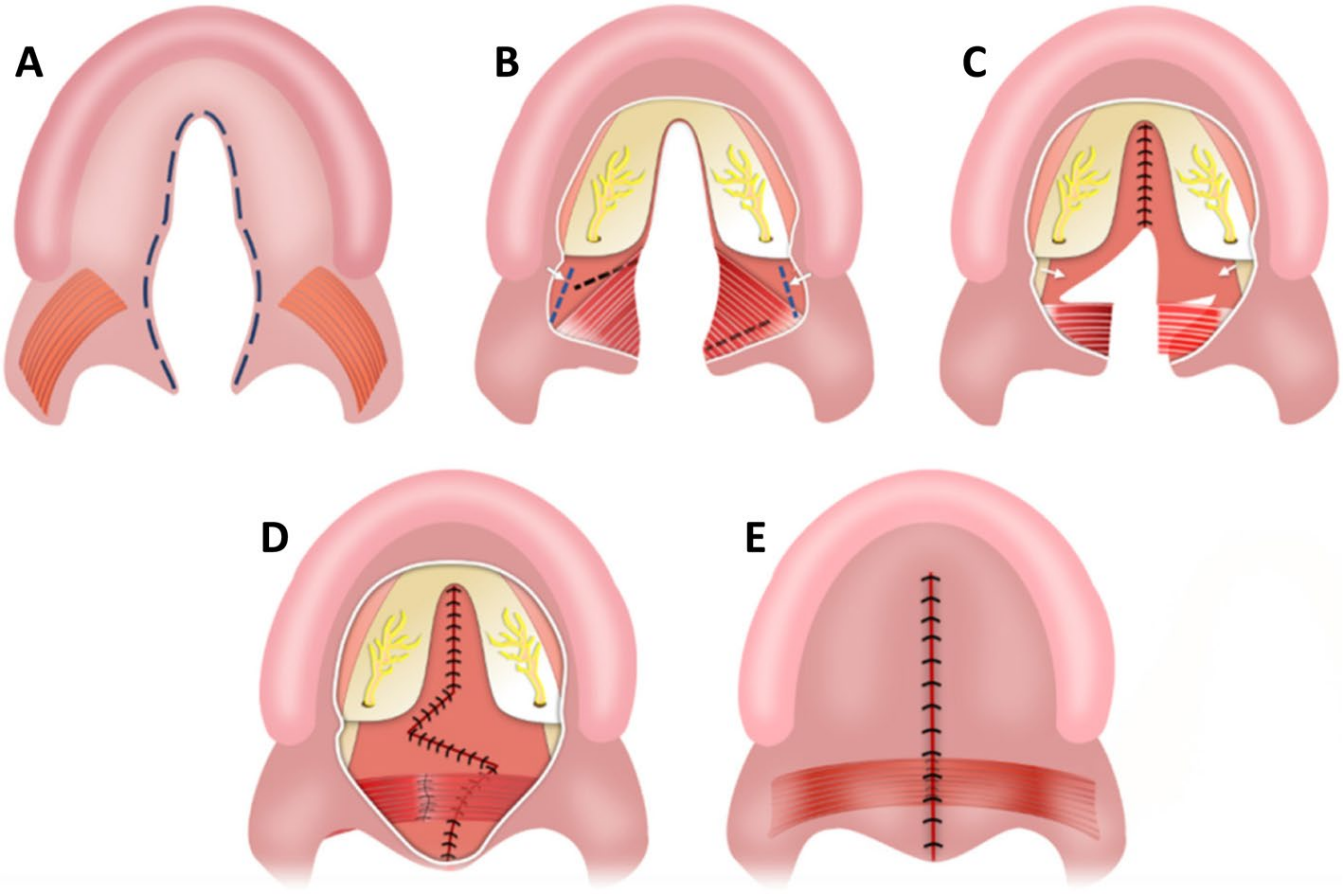
The surgical procedures of palatoplasty using the Sommerlad-Furlow modified technique. **A** an incision was made along the edge of the cleft to separate the oral mucosa layer and nasal mucosa layer. **B** A considerable amount of hard palate mucoperiosteal flap elevation and release of greater palatine neurovascular pedicles, nasopharyngeal incision is made on the medial pterygoid plate using an electrotome. **C** The nasal mucoperiosteum was peeled off anteriorly from the palatine bone and medially from the medial pterygoid plate toward the cranial base and suturing the nasal layer of the hard palate. The nasal musculomucosal layer was subjected to radical muscle dissection. then Z-plasty flaps on the nasal layer of the soft palate were designed. **D** Complete suturing of the nasal layer of soft palate then suturing the dissected palatal muscle. **E** The oral layer is sutured without relaxing incisions

The present S.F. technique was invented based on the principles of both Sommerlad and Furlow techniques. The S–F technique's surgical procedures are summarized as follows (Fig. 1): an incision was made along the edge of the cleft to separate the oral and nasal mucosa (Fig. 1A), then adequate elevation of the oral mucoperiosteal flaps on the hard palate and to release of the greater palatine neurovascular pedicles, making a nasopharyngeal incision on the medial pterygoid plate using an electrotome (Fig. 1B), and the nasal mucoperiosteum was peeled off from the medial pterygoid plate toward the base of the skull and also anteriorly from the palatine bone, the nasal mucosa of the left side underwent radical muscular dissection (Fig. 1C). After suturing the nasal layer of the hard palate, Z-plasty flaps on the nasal layer of the soft palate were designed (Fig. 1C). Complete suturing of the nasal layer of the soft palate, then suturing of the dissected palatal muscular flap to the right myomucosal flap (Fig. 1D), lastly, suturing the oral layer without relaxing the incisions (Fig. 1E).

The Sommerlad technique is summarized as follows [32]: incising the cleft margin, suturing of the nasal mucosa layer, dissecting and retro-positioning the palatal muscles across the posterior part of the velum, and finally suturing the oral mucosa layer.

### Cephalometry

All of the lateral cephalometric radiographs were taken with the same equipment by the same experienced radiologist while the participants were in centric occlusion and a standardized upright position, with the transporionic axis and Frankfort horizontal plane parallel to the surface of the floor [28, 33]. Two well-trained assessors (an oral surgeon, S. Elayah and an orthodontist, W. Al-Gumai) used DOLPHIN Imaging Software (Dolphin Imaging Version 11.95.07.24 Premium, Chatsworth) [34] to trace twice to eliminate measurement errors. All the study variables were measured using stable landmarks, including 12 linear (mm) and 10 angular (°) variants. On each lateral cephalogram, the following landmarks were identified:

Cranial Base; Anterior Cranial Base length, S–N (mm); Posterior Cranial Base length, S-Ba (mm) and Cranial Base Angle, S–N-Ba (°) (Fig. S1).

Maxilla; Maxillary Length, Co-A (mm); Anterior Upper Facial Height, N-ANS (mm); Posterior Upper Facial Height, S- PM (mm); Maxillary Sagittal Position, SNA (°) and Maxillary anteroposterior inclination, SN-PP(°) (Fig. S2).

Mandible: Mandibular length, Co-Gn (mm); Corpus (Body) Length, Go-Gn (mm); Ramus Height, Ar-Go (mm); Mandibular sagittal Position, SNB (°); Total Anterior Facial Height, N-Me (mm); Lower Anterior Facial height, ANS-Me(mm); Total Posterior Facial Height, S-Go (mm) and Mandible anteroposterior inclination, MP-SN(°) (Fig. S3).

Intermaxillary relation; Maxillomandibular differences, Co-Gn—Co-A(mm); Sagittal Intermaxillary Relationship, ANB (°); and Palatal-Mandibular Angle, PP-MP (°) (Fig. S4).

Occlusion; Occlusal Plane to SN Plane, OP-SN (°); Occlusal Plane to FH Plane, OP-FH (°) and Occlusal Plane to Mandibular Plane, OP-MP (°) (Fig. S5).

### Statistical analysis

Statistical Package for Social Sciences (SPSS) version 27(Chicago, USA) was used to compute descriptive and analytical statistics. Kolmogorov–Smirnov confirmed the data to evaluate the normality distribution. Kruskal Wallis H and Mann–Whitney tests were used to assess differences in craniofacial morphology among the three groups. In addition, the inter-observer reliability of measures was analyzed using the intraclass correlation coefficient test (ICC). For all metrics, ICC tests were more than 0.9, indicating an acceptable level of agreement. *P* < 0.05 was regarded to be significant.

## Results

90 participants, 60 patients with non-syndromic cleft palate underwent surgical repair using the S-F technique (30) and S technique (30) with no significant difference found between them regarding cleft width, cleft type, and age at repair (Table 2). While the other 30 were normal participants with skeletal class I pattern, with no significant difference found among groups regarding gender and age at cephalogram collection. The means age at collection of cephalograms were 6.03 ± 0.80 (5–7) in the S group, 5.96 ± 0.76 (5–7) in the S-F group, and 5.91 ± 0.87 (5–7) in the control group (Table 1). Our comparison of maxillofacial morphology among three groups showed in (Table 3).

**Table 2:** Demographic features of both palatoplasty groups

Variables	Sommerlad group	S.Fgroup	*P-value*
**Age at the palatoplasty**, year			
Mean ± SD	1.06 ± 0.36	1.14 ± 0.26	0.18
(Min–Max)	(0.5–1.67)	(0.5–1.67)	
**Cleft width**, mm			
Mean ± SD	9.95 ± 1.92	10.92 ± 2.43	0.11
(Min–Max)	(7–14)	(7–15)	

*S-F* Sommerlad-Furlow modified technique, *SD* Standard deviation

**Table 3:** Results of comparison of maxillofacial morphology between three groups

Variables	Sommerlad group (**I**)	S.F group (**II**)	Control group (**III**)	*P*-value		
	Mean ± SD	Mean ± SD	Mean ± SD	**I** vs **II I** vs **III II** vs **III**		
**Cranial Base**						
S-N^a^	54.7 ± 4.3	55.4 ± 4.3	56.7 ± 4.3	0.67	0.08	0.23
S-Ba^a^	29.2 ± 3.1	32.2 ± 4.1	32.6 ± 4.5	0.01	0.01	0.80
S–N-Ba^b^	131.7 ± 5.2	128.8 ± 6.7	129.4 ± 5.3	0.09	0.11	0.58
**Maxilla**						
Co-A^a^	59.6 ± 5.3	62.3 ± 5.4	65.1 ± 6.3	0.06	< 0.01	0.07
N-ANS^a^	41.0 ± 3.8	42.4 ± 4.6	43.0 ± 4.0	0.46	0.14	0.54
S- PM^a^	29.4 ± 2.8	31.1 ± 4.3	32.6 ± 4.0	0.07	< 0.01	0.07
SNA^b^	75.7 ± 4.7	78.3 ± 5.0	79.4 ± 4.6	0.04	< 0.01	0.42
SN-PP^b^	20.4 ± 4.4	19.6 ± 5.5	17.9 ± 4.2	0.46	0.01	0.09
**Mandible**						
Co-Gn^a^	82.4 ± 5.5	83.0 ± 7.4	85.9 ± 8.2	0.68	0.05	0.16
Go-Gn^a^	61.2 ± 4.9	60.8 ± 6.9	62.6 ± 6.8	0.58	0.28	0.19
Ar-Go^a^	32.0 ± 3.3	32.5 ± 3.9	33.5 ± 4.1	0.59	0.08	0.28
SNB^b^	74.7 ± 3.7	75.3 ± 5.2	76.2 ± 4.5	0.29	0.06	0.44
N-Me^a^	95.4 ± 6.6	93.4 ± 13.3	96.7 ± 7.1	0.58	0.57	0.33
ANS-Me^a^	54.4 ± 5.3	51.0 ± 11.9	53.7 ± 4.4	0.24	0.72	0.42
S-Go^a^	57.7 ± 5.4	56.4 ± 8.7	59.1 ± 6.3	0.75	0.34	0.26
MP-SN^b^	41.1 ± 5.2	42.0 ± 8.3	40.0 ± 6.8	0.99	0.38	0.48
**Intermaxillary relation**						
Co-Gn—Co-A^a^	22.8 ± 3.6	20.1 ± 5.3	20.9 ± 3.5	0.02	0.04	0.51
ANB^b^	1.1 ± 3.4	3.1 ± 1.9	3.3 ± 1.3	0.01	< 0.01	0.64
PP-MP^b^	20.6 ± 6.5	21.4 ± 5.2	22.1 ± 6.1	0.47	0.30	0.41
**Occlusion**						
OP-SN^b^	21.2 ± 8.4	21.0 ± 6.2	20.0 ± 4.4	0.99	0.57	0.48
OP-FH^b^	13.8 ± 9.1	13.4 ± 7.0	12.0 ± 5.1	0.79	0.51	0.55
OP-MP^b^	19.7 ± 8.2	20.1 ± 7.2	20.0 ± 5.7	0.80	0.92	0.77

Significant at the *p* < 0.05 level

*Abbreviations*: *S.F* Sommerlad-Furlow modified technique, *S* sella, *N* nasion, *Ba* Basion, *Co* condylion, *A* A point, *ANS* anterior nasal spine, *PM* pterygomaxillare, *PP* palatal plane, *Gn* Gnathion, *Go* gonion, *B* B point, *Me* menton, *Ar* articular, *MP* Mandibular Plane, *OP* Occlusal Plane, *FH* Frankfort horizontal plane, *SD* standard deviation

^a^Distances between two landmarks were measured in millimeters (mm)

^b^Angles formed by three landmarks were measured in degrees (°)

Regarding cranial base, the results showed that there were no statistically significant differences between the three groups (S, S-F & C) in S–N (54.7 ± 4.3, 55.4 ± 4.3 & 56.7 ± 4.3 and S–N-Ba; 131.7 ± 5.2, 128.8 ± 6.7 & 129.4 ± 5.3) respectively. The S group had a significantly shortest S-Ba than the S-F & C groups (*P* = 0.01), but there was no statistically significant difference between S-F and C groups (*P* = 0.80).

Regarding skeletal maxilla, the S group had significantly shorter Co-A, S- PM and significantly less SNA angle than the C group (*P* =  < 0.01). While there was no significant difference between S-F & C groups (*P* = 0.42). The S group had significantly more SN-PP inclination than the C group (*P* =  < 0.01), with no significant difference between S-F & C groups (*P* = 0.09).

Regarding mandibular bone, there were no statistically significant differences in all linear and angular mandibular measurements between the three groups, except Co-Gn of the S group had significantly shorter length than the C group (*P* = 0.05).

Regarding intermaxillary relation, the S-F group had no significant differences in Co-Gn—Co-A and ANB as compared with the C group. The S group had significantly less ANB angle than S-F & C groups (*P* = 0.01 & *P* =  < 0.01).

Regarding occlusion, there were no significant differences in all angular occlusal measurements between the three groups.

## Discussion

Patients with isolated cleft palate (ICP) should not be included with those with cleft lip and palate (CLP) in scientific studies due to variations in etiology and anatomy. Consequently, scientific studies on patients with clefts should be designed to study subgroups individually [35, 36]. Furthermore, racial factors may play a significant role in cleft palate repair [37], so many studies compare patients with clefts without non-cleft control groups of the same ethnicity [36, 38]. To be more specific and accurate, our study was conducted with patients with the same cleft type ISHCP; participants in three groups were from the same ethnicity.

Our current study assessed the influence of the S-F technique on maxillofacial growth in patients with isolated cleft palate and compared it with the S technique. The anterior cranial base length and angle values in S-F group were closer to C group than the S group without a statistically significant difference. While the S group had significantly shortest posterior cranial base than the S-F & C groups with no statistically significant difference between S-F and C groups. Kulewicz et al. [39] found that the palatoplasty did not significantly affect the growth of the anterior cranial base length. While Liao et al. [40] reported that the stage of palate repair had a significant effect on the means of the length of the posterior cranial base (S-Ba) (*p* = 0.05). As well as, a systematic review concluded that the posterior cranial base is not totally stable, as its dimensions change throughout craniofacial growth and a minor dimensional change is observed even in late adulthood [41]. Some studies hypothesized that the shorter cranial base length in bilateral cleft lip and palate patients was likely caused by early growth retardation and caught-up growth in adulthood [34].

While comparing the measurements of the maxilla, the S.F technique had slightly affected the maxillary measurements, which are insignificant as compared with the C group, but the maxillary length, posterior upper facial height, angle of maxillary sagittal position, and maxillary anteroposterior inclination were significantly affected by S technique. The minimal incision technique in Karsten’s study [42] resulted in better growth of Maxilla. Compared to the Von Langenbeck technique, the isolated cleft palate repair that uses the Sommerlad technique has the advantages of less damage and less tissue scarring while showing no inhibition on the growth of the maxilla [43]. On the other hand, Shibasaki et al. [44] came to the conclusion that treated isolated cleft palate patients had maxillary underdevelopment but with adequate facial balance as a result of positional alterations of the mandible. Recently, Vitali Azouz et al. [45] concluded that there was a low incidence of maxillary hypoplasia after isolated cleft palate repair.

Regarding the mandible, there were no statistically significant differences in all linear and angular mandibular measurements between the three groups except the mandibular length in the S group; it had a significantly shortest length than S.F & C groups. Our results support previous studies, which found that the hard palate repair had no noticeable effect on the mandible's protrusion or the mandibular plane inclination [29, 46, 47]. On the other hand, Shibasaki and Ross [44] reported that the mandible is of normal length but retro-positioned due to the functional response of the mandible to the altered maxilla. This may explain why the S group's mandibular length was shorter than the S-F group.

Regarding the intermaxillary relation, the S-F group had no significant differences in an intermaxillary relationship compared to the C group. The S group had significantly less sagittal intermaxillary angle than S-F & C groups. Some studies [29, 46] reported that the palatoplasty did not significantly affect jaw relation (ANB), whereas another study [39] reported that it did. The influence of the palatoplasty technique has been limited to the transverse development of the maxillary dental arch [48]. Da Silva et al. [49] the intermaxillary relationship was regarded as satisfactory after the primary palatoplasty. On the other hand, more palatal scar tissue from the technique may have a more significant effect on the teeth and the alveolar process than on maxillary growth [29]. Similarly, Karsten et al. [42] reported that a minimal incision technique resulted in better development of the maxilla with better dental occlusion than the Veau–Wardill–Kilner technique, which is claimed to produce relatively large areas of denuded palatal bone.

Scarred palatal mucosa may partially resist further growth if there is tissue undermining and hamulus fracture in the area of the pterygopalatomaxillary junction during the palatal repair.

Overall, the current favorable outcomes observed in both primary palatoplasty techniques may be clarified through the conclusion of two systematic review studies; it is widely accepted that cleft lip repair could have a negative effect on maxillofacial growth; therefore, lip closure is the most important factor in restricting of maxillary growth in patients with UCLP [50, 51]. However, tension from upper lip closure causes retro-inclined upper incisors, a retruded maxilla, and an obtuse nasolabial angle [52]. Typically, this results in an anterior crossbite [53].

The favorable outcomes observed in the S-F technique may be attributed to the three concepts that the S-F technique designed to close the cleft palate under palatal muscle reconstruction using Sommerlad muscle dissection, decreasing the pharyngeal cavity by nasal Z-plasty and a novel incision on the medial pterygoid plate's surface which was designed to make the S-F technique applicable in wider clefts without relaxing incision on the hard palate [27]. In contrast, the Sommerlad technique does not use of Z-plasty flaps, which may result in tension and growth limitation.

The outcomes associated with this study may have been impacted by its limitations. The groups were assessed before puberty. Another limitation was that the enrolled patients were not from a single surgeon. However, both surgeons in the present study had more than 12 years of experience and worked in almost one team. Further studies with large size samples after growth complete will be required for better evaluation and understanding of craniofacial morphology of ICP.

## Conclusion

As a preliminary report, Sommerlad-Furlow modified technique showed maxillary positioning in the face tended to be better, and the intermaxillary relationship was more satisfactory than that in Sommerlad technique when compared them in healthy noncleft participants. While current study has shed light on the effects of cleft palate repair techniques on the maxillofacial growth before puberty, the dynamic nature of skeletal growth necessitates a more extended observation period.

### Supplementary Information

**Additional file 1: Figure S1.** Cranial Base measurements; Anterior cranial base length (S-N, Sella-Nasion); Posterior cranial base length (S-Ba, Sella- Basion); Cranial base angle (S-N-Ba, Sella-Nasion-Basion angle). **Figure S2.** Maxilla measurements; Maxillary Length (Co-A, condylion - A point); Anterior Upper Facial Height (N-ANS, Nasion- anterior nasal spine); Posterior Upper Facial Height (S- PNS, Sella - posterior nasal spine); Sagittal Maxillary Position (SNA, Sella-Nasion- A point angle), and Maxillary Anteroposterior Inclination (SN-PP, Sella-Nasion line- palatal plane angle). **Figure S3.** Mandible measurements; Mandibular Length (Co-Gn, condylion- Gnathion); Corpus (Body) Length (Go-Gn, gonion -Gnathion); Ramus Height (Ar-Go, articular- gonion); Mandibular sagittal Position (SNB, Sella-Nasion- B point angle); Total Anterior Facial Height (N-Me, Nasion- mention); Lower Anterior Facial Height (ANS-Me, anterior nasal spine -mention), Posterior Total Facial Height (S-Go, Sella- gonion) and Mandibular Anteroposterior Inclination (MP – SN, mandibular plane- Sella Nasion line angle). **Figure S4.** Intermaxillary relation measurements; Maxillo-mandibular differences (Co-Gn - Co-A, condylion- Gnathion- condylion - articular); Sagittal intermaxillary relationship (ANB, A point -Nasion - B point angle) and Palatal plane - mandibular plane (PP-MP,) angle. **Figure S5.** Occlusion measurements; Occlusal plane to anterior cranial base angle (OP-SN, Occlusal plane- Sella Nasion line angle); Occlusal Plane to Frankfort horizontal plane angle (OP-FH) angle, and Occlusal plane to mandibular plane (OP-MP) angle.

## Data Availability

The datasets used and analyzed during the study are available from the corresponding author upon reasonable request.
