# Supplementary material for: A comparison of maxillofacial growth in Chinese children with isolated cleft palate treated with two different palatoplasty techniques without relaxing incisions: a preliminary study
Source: BMC Oral Health. 2023 Nov 23;23:914. doi: 10.1186/s12903-023-03588-6 (PMC10668460; doi:10.1186/s12903-023-03588-6)

**Supplementary figures legend**


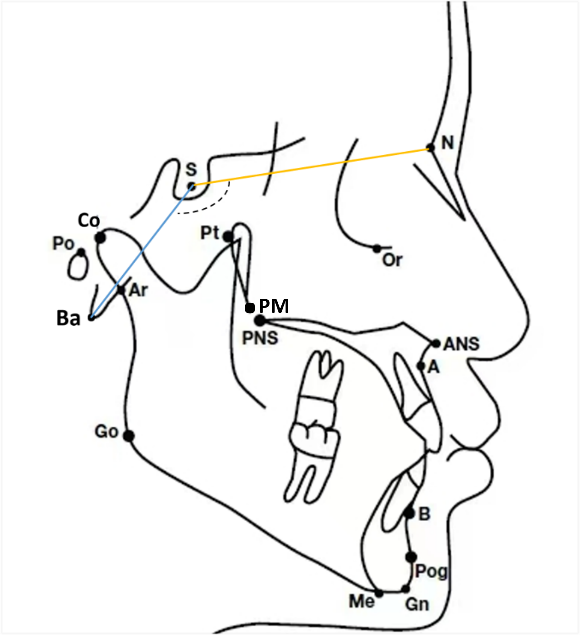
**Fig.S1;** **Cranial Base measurements;** Anterior cranial base length (S-N, Sella-Nasion); Posterior cranial base length (S-Ba, Sella- Basion); Cranial base angle (S-N-Ba, Sella-Nasion-Basion angle).


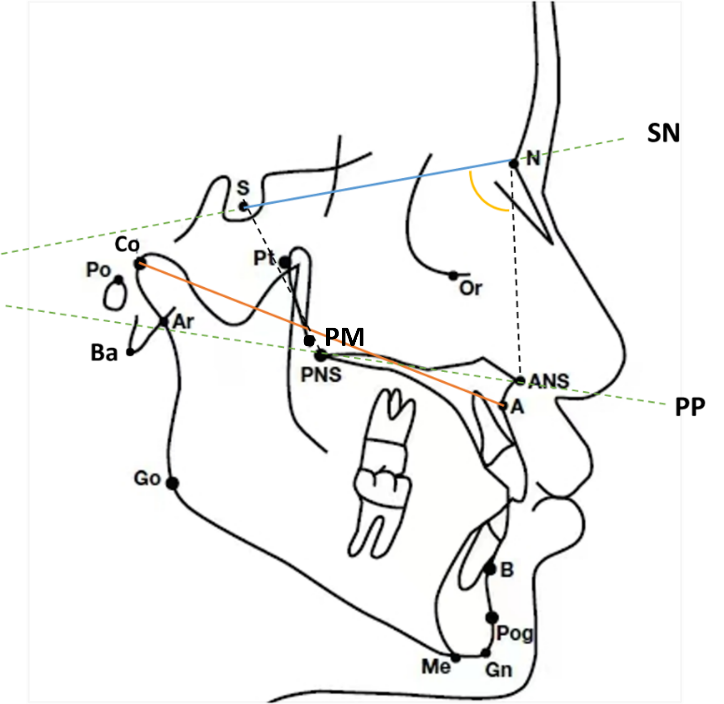
**Fig.S2; Maxilla measurements;** Maxillary Length (Co-A, condylion - A point); Anterior Upper Facial Height (N-ANS, Nasion- anterior nasal spine); Posterior Upper Facial Height (S- PNS, Sella - posterior nasal spine); Sagittal Maxillary Position (SNA, Sella-Nasion- A point angle), and Maxillary Anteroposterior Inclination (SN-PP, Sella-Nasion line- palatal plane angle).

**Fig.S3 Mandible measurements;** Mandibular Length (Co-Gn, condylion- Gnathion); Corpus (Body) Length (Go-Gn, gonion -Gnathion); Ramus Height (Ar-Go, articular- gonion); Mandibular sagittal Position (SNB, Sella-Nasion- B point angle); Total Anterior Facial Height (N-Me, Nasion- mention); Lower Anterior Facial Height (ANS-Me, anterior nasal spine -mention), Posterior Total Facial Height (S-Go, Sella- gonion) and Mandibular Anteroposterior Inclination (MP – SN, mandibular plane- Sella Nasion line angle).


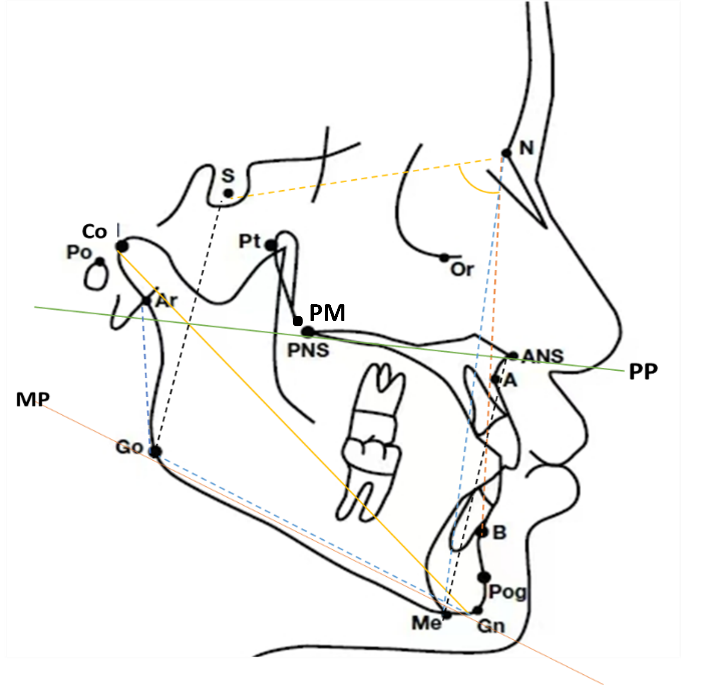


**Fig.S4; Intermaxillary relation measurements;** Maxillo-mandibular differences (Co-Gn - Co-A, condylion- Gnathion- condylion - articular); Sagittal intermaxillary relationship (ANB, A point -Nasion - B point angle) and Palatal plane - mandibular plane (PP-MP,) angle.


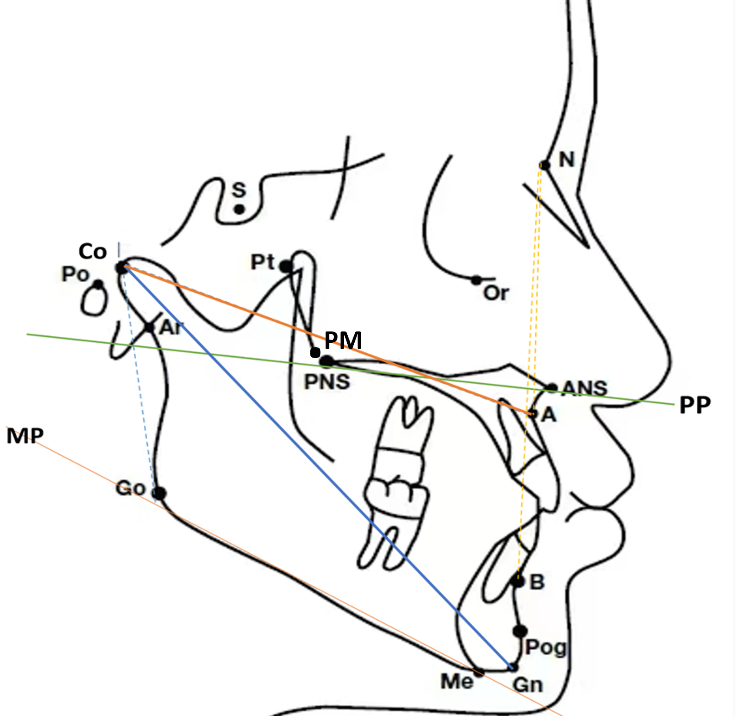


**Fig.S5; Occlusion measurements;** Occlusal plane to anterior cranial base angle (OP-SN, Occlusal plane- Sella Nasion line angle); Occlusal Plane to Frankfort horizontal plane angle (OP-FH) angle, and Occlusal plane to mandibular plane (OP-MP) angle.


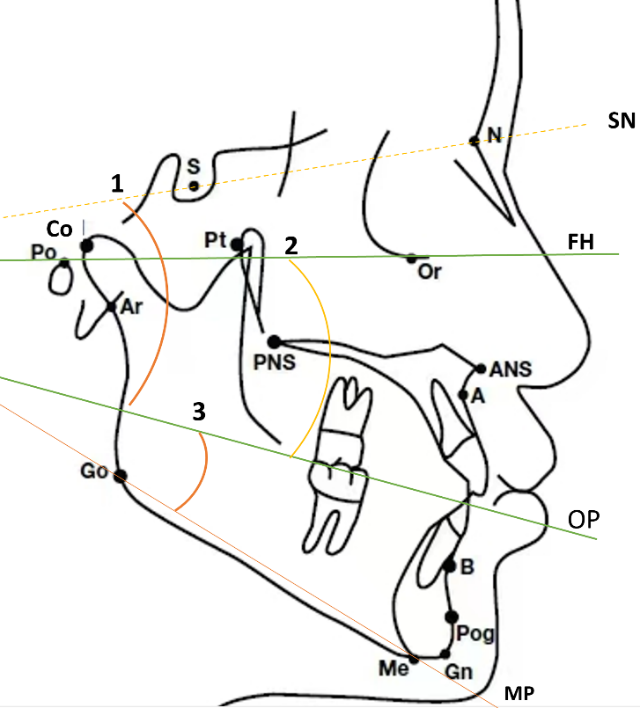

Supplement: Supplementary file 1 — Additional file 1: Figure S1. Cranial Base measurements; Anterior cranial base length (S-N, Sella-Nasion); Posterior cranial base length (S-Ba, Sella- Basion); Cranial base angle (S-N-Ba, Sella-Nasion-Basion angle). Figure S2. Maxilla measurements; Maxillary Length (Co-A, condylion - A point); Anterior Upper Facial Height (N-ANS, Nasion- anterior nasal spine); Posterior Upper Facial Height (S- PNS, Sella - posterior nasal spine); Sagittal Maxillary Position (SNA, Sella-Nasion- A point angle), and Maxillary Anteroposterior Inclination (SN-PP, Sella-Nasion line- palatal plane angle). Figure S3. Mandible measurements; Mandibular Length (Co-Gn, condylion- Gnathion); Corpus (Body) Length (Go-Gn, gonion -Gnathion); Ramus Height (Ar-Go, articular- gonion); Mandibular sagittal Position (SNB, Sella-Nasion- B point angle); Total Anterior Facial Height (N-Me, Nasion- mention); Lower Anterior Facial Height (ANS-Me, anterior nasal spine -mention), Posterior Total Facial Height (S-Go, Sella- gonion) and Mandibular Anteroposterior Inclination (MP – SN, mandibular plane- Sella Nasion line angle). Figure S4. Intermaxillary relation measurements; Maxillo-mandibular differences (Co-Gn - Co-A, condylion- Gnathion- condylion - articular); Sagittal intermaxillary relationship (ANB, A point -Nasion - B point angle) and Palatal plane - mandibular plane (PP-MP,) angle. Figure S5. Occlusion measurements; Occlusal plane to anterior cranial base angle (OP-SN, Occlusal plane- Sella Nasion line angle); Occlusal Plane to Frankfort horizontal plane angle (OP-FH) angle, and Occlusal plane to mandibular plane (OP-MP) angle. [file 12903_2023_3588_MOESM1_ESM.docx]
